# Impact of HER2 status in resected gastric or gastroesophageal junction adenocarcinoma in a Western population

**DOI:** 10.3332/ecancer.2020.1020

**Published:** 2020-03-24

**Authors:** Nieves Martínez Lago, María Vieito Villar, Rafael Varela Ponte, Ihab Abdulkader Nallib, Juan José Carrera Alvarez, José Ramón Antúnez López, Rafael López López, María Elena Padin Iruegas

**Affiliations:** 1 Medical Oncology Department, University Hospital A Coruña, 15006 A Coruña, Spain; 2 GU, Sarcoma and Neuro-oncology Unit, Vall d’Hebron University Hospital, 08035 Barcelona, Spain; 3 Radiology Department, University Hospital Complex of Santiago de Compostela, 15706 Santiago de Compostela, Spain; 4 Pathology Department, University Hospital Complex of Santiago de Compostela, 15706 Santiago de Compostela, Spain; 5 Medical Oncology Department, University Hospital Complex of Santiago de Compostela, 15706 Santiago de Compostela, Spain; 6 Human Anatomy and Embryology Area, Faculty of Physiotherapy, Department of Functional Biology and Health Sciences, Vigo University, 36310 Vigo, Pontevedra, Spain

**Keywords:** HER2, gastric cancer, prognosis, immunohistochemistry, DuoCISH

## Abstract

**Background:**

HER2 status is a predictive biomarker of response to trastuzumab in advanced gastric or gastroesophageal junction (GEJ) adenocarcinoma. However, there is relatively little known about the role of HER2 in resected gastric or GEJ adenocarcinoma in the Western population.

**Methods:**

Retrospective, observational, single centre study of patients with gastric or GEJ adenocarcinoma undergoing surgery with curative intent between January 2007 and June 2014 in the University Hospital Complex of Santiago de Compostela. The expression of HER2 was determined by immunohistochemistry (IHC) using DAKO-HercepTest™ and gene amplification with DuoCISH using a DAKO-DuoCISH kit. The study of HER2 expression and amplification was carried out in all the patients and it was correlated with classic clinicopathological parameters, survival and recurrence pattern.

**Results:**

106 patients were included. HER2 expression was as follows: 71.7% HER2 negative, 21.7% HER2 equivocal and 6.6% HER2 positive, or with HER2 overexpression. 13.2% of patients (14/106) had HER2 amplification by DuoCISH. A significant association was seen between overexpression and amplification of HER2 (*p* < 0.001).

HER2 positivity was associated with the intestinal subtype (*p* = 0.010) and a low grade of differentiation (*p* = 0.018). Likewise, HER2 was significantly associated with a worse prognosis: overall survival (OS) 32.3 months HER2 positive versus 93.9 months HER2 negative (HR 0.42; confidence interval 95% 0.18–0.93; *p* = 0.028); and the presence of distant metastasis without accompanying locoregional recurrence (*p* = 0.048).

**Conclusion:**

HER2 status defines a subgroup with differentiated clinicopathological characteristics, worse prognosis and distant dissemination, without accompanying locoregional recurrence, in patients with resected gastric or GEJ adenocarcinoma operated on in a Western population.

## Background

Gastric cancer is the fifth most common neoplasm and the third cause of death from cancer worldwide [1]. Surgery is the treatment of choice in resectable gastric adenocarcinoma. However, despite the advances made in surgical treatment in recent years, surgery continues to be curative only in the early stages. In locally advanced resectable tumours, 5-year survival rates remain poor, ranging between 10% and 50%, so complementary treatment strategies are needed [2].

Various strategies, such as perioperative chemotherapy, adjuvant chemotherapy or chemoradiotherapy have been tested with an absolute benefit in terms of OS around 10%–15%, but to date there is no unanimity on which strategy to use [3–6].

The HER2/neu oncogene is located on the short arm of chromosome 17 and encodes the 185 kDa HER2 protein, a transmembrane receptor formed by an extracellular portion of ligand binding, a transmembrane domain, and an intracellular portion with tyrosine kinase activity. The HER2 receptor has been widely studied, first in breast cancer and more recently in gastric cancer [7].

Overexpression and/or amplification of HER2 has been described in 10%–15% of patients with advanced gastric or gastroesophageal junction (GEJ) adenocarcinoma and has been demonstrated to be predictive of the response to chemotherapy and trastuzumab [8, 9].

Thus, in the ToGA study, a Phase III randomised study showed the benefit, in terms of OS, progression-free survival (PFS) and response rate, of adding trastuzumab to first-line chemotherapy treatment based on cisplatin and a fluoropyrimidine in patients with intense overexpression and/or amplification of HER2. However, the benefit of other anti-HER2 drugs such as lapatinib, Ado-trastuzumab emtansine (TDM-1) or pertuzumab, could not be demonstrated, nor the benefit of continuing with anti-HER2 therapy after progression [10–13].

In contrast, in localised gastric cancer, the role of HER2 remains controversial. Thus, while in the Asian population, HER2 positivity has been shown to be a poor prognostic factor, in the Western population, its role has not yet been clarified. Thus, due to the scarce data, we have on the western population and its heterogeneity, with simultaneous inclusion of resected and metastatic patients, of gastric, GEJ or even oesophageal carcinomas, as well as the use of different methodologies for their evaluation and interpretation, which in some cases include immunohistochemistry (IHC) techniques exclusively, while others are complemented by hybridisation techniques, the results have been very disparate and difficult to compare [14].

For this reason, the hypothesis of our study was that the HER2 status defines a subgroup of patients with resected gastric or GEJ adenocarcinoma in the Western population, with differentiated clinicopathological characteristics, worse prognosis and a distinct pattern of recurrence.

## Methods

We conducted a retrospective, observational, single centre study of patients with a confirmed diagnosis of gastric or GEJ adenocarcinoma, who underwent surgery with curative intent between January 2007 and June 2014 at the University Hospital Complex of Santiago de Compostela (Spain) and whose histological samples were stored in the biobank of said complex.

Patients with a history of neoplasms within the previous 5 years were excluded, as well as those who had received cancer treatment prior to surgery, those who had died in the immediate postoperative period (defined as 60 days after surgery), and those who lacked histological material or had incomplete clinicopathological or follow up data.

The study was approved by the relevant Research Ethics Committee, and conducted in accordance with the ethics standards of the Declaration of Helsinki. All patients had given their prior consent for the inclusion of samples in the biobank of the University Hospital Complex of Santiago de Compostela and for secondary studies to be carried out with these samples.

HER2 determination was performed on samples from surgical resection. First, before performing IHC and *in situ* hybridisation techniques, all available histological preparations were evaluated, selecting representative samples of the neoplasm and avoiding hypocellular areas, areas with necrosis or haemorrhage, poor tissue quality, autolysis or artifacts. In the case of mixed tumours, areas with intestinal subtype histology were selected.

### Determination of HER2 protein expression via IHC

The study of HER2 protein expression was performed by means of IHC techniques using the FDA-approved kit for the determination of HER2 in advanced gastric carcinoma, the Herceptest™ Kit (Dako, Carpinteria, California, USA). The procedure was carried out according to the manufacturer instructions. The evaluation was carried out by two observers independently, according to the Rüschoff/Hoffman methodology for surgical sampling (Table 1). In discordant cases, an interobserver agreement was made for the final statistical analysis.

### Determination of HER2 gene amplification by DuoCISH

HER2 amplification was determined by the DuoCISH technique, using the HER2 FISH kits pharmDx™ and DuoCISH™ (Dako, Carpinteria, California USA). This determination was made in all patients, regardless of the results of HER2 expression. The procedure was carried out according to the manufacturer’s instructions. For the evaluation, 20 nuclei were selected and the HER2 and CEN-17 signals were quantified, calculating the HER2/CEN-17 ratio. HER2 was considered amplified or positive if the HER2/CEN-17 ratio was >2, and non-amplified or negative if HER2/CEN-17 <2.

### Statistical analysis

The statistical analysis was carried out using SPSS 19.0 statistical software. A descriptive analysis of qualitative and quantitative variables was carried out, utilising an analysis of frequencies or percentages in the case of qualitative and quantitative variables and determining the mean, median and range in the case of quantitative variables. Comparisons of categorical variables were analysed using either the Chi-square test or Fisher’s exact test based on sample size. The comparison of independent groups, with respect to quantitative variables, was carried out by applying the *T*-Student or analysis of variance test if the variable followed a normal distribution and the non-parametric Wilcoxon–Mann–Whitney or Kruskall–Wallis test, if it did not follow the normal distribution.

With regard to survival data, we define OS as the interval between the date of surgery and the date of death from any cause or loss to follow up, and specific OS for gastric carcinoma as the interval from the date of surgery to the date of death from this pathology, with patients dying from other causes being suppressed.

A Kaplan–Meier model was analysed for OS and specific OS, estimating the median and 95% confidence interval (95% CI). The differences in the survival curves were compared by using the Log-Rank test. In all analyses, a bilateral risk, or significance level *p* = 0.05, was established.

## Results

### Population characteristics

106 patients were included. The main clinicopathological characteristics are detailed in Table 2. 64.2% of the patients were male and the median age was 69 years (range of 38–81 years). 66% of the tumours were localised in the distal stomach, 72.6% were Lauren’s histological subtype and 60.3% were low-grade tumours. The median number of resected adenopathies was 24 (range 3–65) and 83% resected >15 adenopathies, with D2 lymphadenectomy performed in 58.5% of the patients. 59% of patients had locally advanced tumours (T3-4) and lymph node involvement in 54.7%. 56.6% were treated with adjuvant radiochemotherapy.

### HER2 expression and amplification

**Figure 1.**
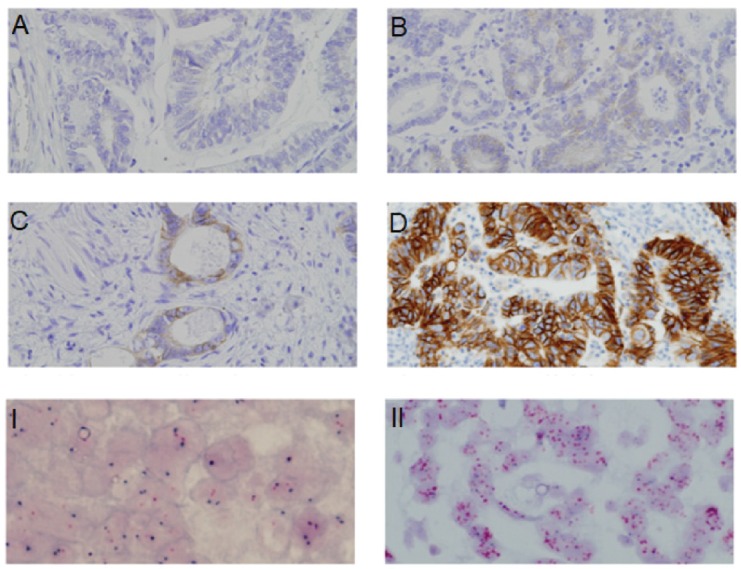
HER2 expression through IHC. (A): Absent expression (HER2 0+). (B): Weak expression (HER2 1+). (C): Moderate expression (HER2 2+). (D): Strong expression (HER2 3+). I. HER2 not amplified. II. HER2 amplified.

**Figure 2.**
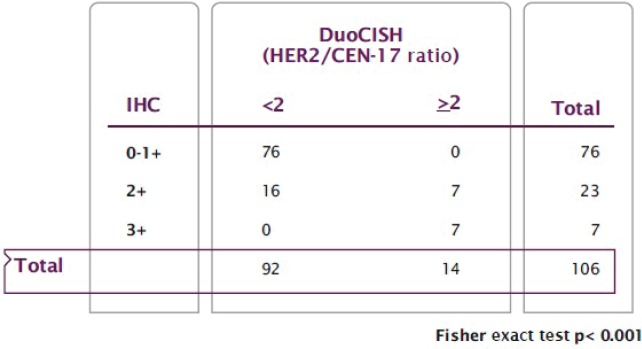
Relationship between HER2 expression (IHC) and HER2 amplification (DuoCISH).

With regard to HER2 expression, 60 samples (56.6%) had no expression and were classified as 0+, 16 (15.1%) had weak expression or 1+; 23 patients (21.7%) had moderate expression or 2+, and the remaining 7 (6.6%) had strong expression or 3+. Therefore, according to Hoffman’s classification, 76 patients (71.7%) were classified as HER2 negative, 7 (6.6%) as positive, and the remaining 23 (21.7%) as equivocal (Figure 1). Subsequently, hybridisation techniques were performed in the 106 patients (100%) included in the study, and HER2 amplification was observed in 14 patients (13.2%); 7 patients with intense or 3+ HER2 expression (100%), 7 (30.4%) with moderate or 2+ expression and none (0%) with absent or weak expression (0.1+). A statistically significant association between expression and HER2 amplification was identified (*p* < 0.001) (Figure 2).

### HER2 and its association with classic clinicopathological parameters

HER2 positivity was significantly associated with Lauren intestinal subtype (100% versus 68.5%, *p* = 0.010), low-grade tumours, defined as G1–G2, (92.9% versus, 55.4%, *p* = 0.008) and less deep tumour invasion, defined as T1–T2 (71.4% versus 35.9%, *p* = 0.018), with no significant difference in lymph node involvement (64.3% versus 53.3%, *p* = 0.568). Additionally, there was a trend towards a non-significant association between HER2 positivity and proximal location tumours (42.9% versus 31.5%, *p* = 0.074). However, no relationship was observed between HER2 positivity and the age at diagnosis (*p* = 0.753), gender (*p* = 370), lymphovascular invasion (*p* = 539) or perineural invasion (*p* = 0.776) (Table 3).

### HER2 and impact on survival

**Figure 3.**
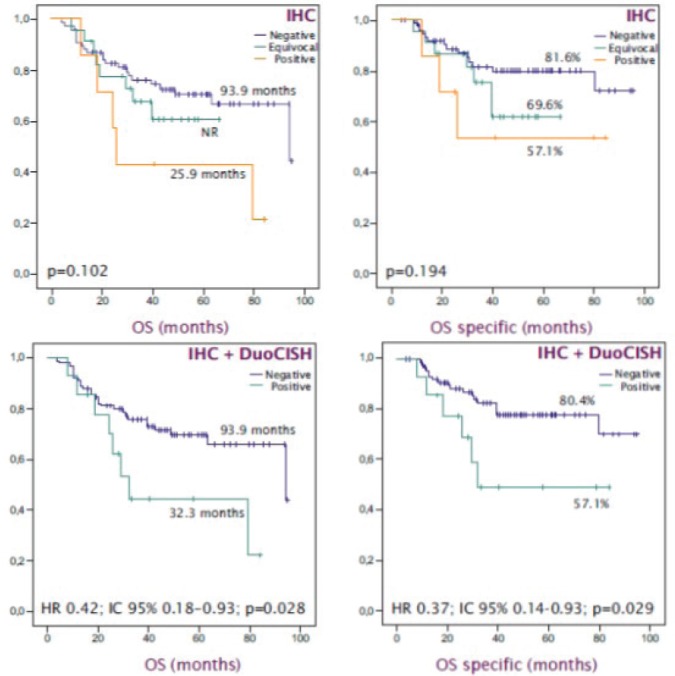
OS-specific Kaplan -Meier curves to estimate the prognostic impact of HER2 status. (A): OS according HER2 expression. (B): OS specific according HER2 expression. (C): OS based on HER2 status. (D): OS specific based on HER2 status.

With a median follow up of 54 months, the median OS was 93.9 months. HER2 expression was not significantly associated with a worse prognosis: median OS HER2 was negative/equivocal/positive at 93.9 months versus 25.9 months (*p* = 0.102), trend maintained in specific OS (*p* = 0.194). HER2 positivity, combining expression and amplification, was significantly associated with worse survival: OS 32.3 months HER2 positive versus 93.9 months HER2 negative (HR 0.42; 95% CI 0.18 – 0.93 Log Rank *p* = 0.028); which was confirmed in the OS analysis specific to the gastric carcinoma (HR 0.37; IC 95% 0.14–0.93 Log Rank *p* = 0.029) (Figure 3).

### HER2 and recurrence pattern

**Figure 4.**
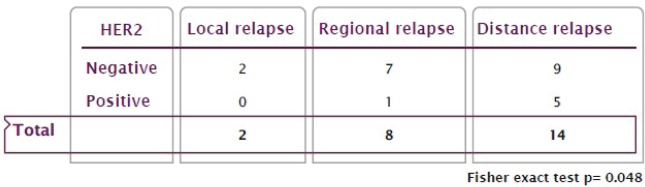
Recurrence pattern based on HER2 status.

30.2% of patients presented with tumour recurrence, with a significant association between HER2 positivity and tumour recurrence (42.8% versus 19.6%, *p* = 0.049). 58.3% of patients with distant metastases identified a significant association between HER2 positivity and distant metastases without accompanying local or peritoneal recurrence (83.3% versus 38.9%, *p* = 0.048) (Figure 4).

## Discussion

The use of trastuzumab in advanced, HER2-positive gastric or GEJ cancer, has been the first successful example of personalised medicine in gastroesophageal carcinoma. However, results from other studies of drugs that target the HER2 pathway have been disappointing. It is, thus, crucial to understand the role this pathway plays in the tumourigenesis of gastric carcinoma, starting with its role in localised disease.

We, therefore, analysed in our study the role of HER2 status in 106 patients who underwent radical surgery. Our aim was to determine if these patients defined a subgroup with differentiated clinicopathologic characteristics, prognostic impact and differentiated dissemination and/or recurrence patterns.

To begin with, we began by analysing the clinicopathologic characteristics of our population. The median age at diagnosis was 70 to 80, the male-to-female ratio was 2:1, and 10% of tumours were located in the GEJ. Tumours were also predominantly of the intestinal Lauren subtype, which was consistent with earlier reports [9].

HER2 status testing in our study identified 14 HER2-positive patients (13.2%). This percentage is consistent with previously published studies conducted in Western populations with advanced disease. Those studies also combined protein expression and gene amplification techniques, and found HER2-positivity that ranged from 10% to 20% [9, 15, 16].

The methodology for determining HER2 status in our study combined IHC techniques to determine protein expression with hybridisation techniques (DuoCISH) for gene amplification. Both techniques have FDA approval and have been standardised in normal clinical practice. All samples were also evaluated by two independent pathologists, pursuant to the main consensus guidelines for HER2 determination and interpretation in advanced gastric carcinoma. The only exception to these guidelines was that hybridisation techniques were performed in all patients, and not only for those with moderate or equivocal expression. The aim of this practice was to identify possible discordant cases, such as the presence of negative, amplified tumours or vice-versa [17, 18].

In our study, 100% of patients with strong expression (3+), and 0% of patients with absent/weak expression (0-1+), presented gene amplification. We, thus, found 100% concordance between the two diagnostic techniques we used. However, only 30.4% of samples with moderate expression (2+), classified as equivocal, showed amplification, a result consistent with earlier studies of advanced disease. The main consensus guidelines state that performing hybridisation techniques is mandatory in this circumstance [9, 15, 16].

These results would confirm the validity of the HER2 algorithm for use in localised populations with advanced disease. IHC techniques are the first option for all patients in these groups, with hybridisation techniques reserved for patients with moderate or equivocal expression. This distinction minimises the cost and time required for HER2 determination.

There is another notable characteristic that we have observed for the first time in a Western European population that had undergone resection. We found an association between HER2 positivity and classic clinicopathologic parameters identified previously in advanced disease, such as the Lauren classification intestinal subtype and proximal location. We also found a better degree of differentiation, observed in an earlier meta-analysis of HER2 impact in a mainly Asian population.

Finally, we have corroborated, for the first time in a Western European population that had undergone resection that HER2 amplification leads to a higher percentage of recurrence, and thus significantly lower OS. We have also documented a characteristic pattern of recurrence in the HER2-positive population. This pattern consistently involves metastasis within the liver or further from the peritoneal cavity, without local recurrence or accompanying peritoneal carcinomatosis [9, 16, 19].

All of these findings suggest to us how aggressive this tumour subtype is, and are consistent with earlier studies in breast cancer. Those studies found that HER2-positive tumours were related with a worse prognosis and a higher recurrence rate, chiefly in the viscera. These studies also found that luminal subtype tumours were characterised by better survival and associated with recurrence in bone level.

HER2 profiles a subgroup with differentiated characteristics, chiefly related to prognosis, percentage and recurrence patterns. These characteristics could have implications that are therapeutic and not just prognostic. Those implications involve classic supplementary treatment strategies. For example, adjuvant radio chemotherapy has shown benefits for recurrence rates, essentially reducing locoregional recurrences, but not affecting more distant recurrence. This strategy may not be useful in HER2-positive populations, as observed in the unplanned retrospective analysis from the INT0116 study, which was based on HER2 status. That analysis found that benefits were limited to the HER2-negative population [20].

In addition to helping us prioritise types of adjuvant treatment, HER2 could be a target for reducing relapse percentages. It could also raise response rates by adding anti-HER2 therapies to neoadjuvant treatment.

We currently have the results of two small, phase II studies of perioperative chemotherapy, the NEOHX and HER-FLOT studies, in which trastuzumab was added to a CAPOX or FLOT regimen, respectively. Rates of pathological complete response were 8.3% and 22.2%, respectively, without significant increases in toxicity or surgical complications [21, 22].

Moreover, the TOXAG study showed that adding trastuzumab to adjuvant radio chemotherapy is tolerable in terms of toxicity, though we currently lack data on its effectiveness [23].

Two randomised, phase III studies to evaluate the benefits of anti-HER2 therapy combined with perioperative chemotherapy in gastric or locally advanced GEJ adenocarcinoma are underway, INNOVATION and PETRARCA. Their results will help us understand the predictive value of HER2 in resectable populations, as well as its impact on resection and survival rates in these patients [24, 25].

## Conclusion

We have corroborated in this study the validity of the algorithm for determining HER2 status in advanced disease for application in gastric adenocarcinoma or resected GEJ adenocarcinoma. We have also shown an association between HER2 positive and classic clinicopathologic characteristics, such as intestinal Lauren subtype, proximal location and low-grade tumours (G1-2). We have also confirmed the negative prognostic impact of HER2 positivity and the presence of distant recurrences without accompanying locoregional recurrence. The presence of HER2 amplification thus plays a role in the pathogenesis of gastric cancer, starting in the earliest stages. HER2 amplification also determines a series of molecular characteristics that produce a differentiated phenotype with a poor prognosis.

## List of abbreviations

IHC: immunohistochemlstry
OS: overall survival
GEJ: gastroesophageal junction

## Conflicts of interest

The authors declare that there are no conflicts of interest.

## Authors’ contributions

All authors have made substantial intellectual contributions to this article.

## Funding

The authors received no specific funding for this work.

## Figures and Tables

**Table 1.** Rüschoff/Hoffman criteria for the evaluation of HER2 expression by IHC in gastric or GEJ adenocarcinoma in surgical specimen.

Score	Description	Result
0	Absence of staining or staining in less than 10% of the cells	Negative
1	Membrane staining, at least laterally, was almost imperceptible in at least 10% of the cells	Negative
2	Membrane staining, at least laterally, was moderate in at least 10% of the cells	Equivocal
3	Membrane staining, at least laterally, was heavy in at least 10% of the cells	Positive

**Table 2.** Population characteristics.

Characteristics	N (%)
Age Median (Range)	69 (38–81)
Gender Male Female	68 (64.2) 38 (35.8)
Tumour location Proximal Distal Unknown	35 (33.0) 70 (66.0) 1 (1.0)
Lauren histological subtype Intestinal Diffuse Mixed	77 (72.6) 25 (23.6) 4 (3.8)
Histological grade Low Grade (G1–G2) High degree (G3–G4)	64 (60.4) 42 (39.6)
Lymphadenectomy D0-1 D2	44 (41.5) 62 (58.5)
Resected adenopathies <15 >15	18 (17.0) 88 (83.0)
Tumour invasion (T) T1–T2 T3–T4	43 (40.6) 64 (59.4)
Lymph node involvement (N) N0 N1-3	48 (45.3) 58 (54.7)
Lymphovascular invasion No Yes	48 (45.3) 58 (54.7)
Perineural invasion No Yes	52 (49.1) 54 (50.9)
Adjuvant therapy No Yes	46 (43.4) 60 (56.6)

**Table 3.** HER2 and classic clinicopathological features.

Characteristics	HER2 Negative 92 (86.7%)	HER2 Positive 14 (13.2%)	*p*
Age >65 years <65 years	64 (69.6) 28 (30.4)	11 (78.6) 3 (21.4)	0.490
Gender Male Female	57 (62.0) 35 (38.0)	11 (78.6) 3 (21.4)	0.227
Tumour location Proximal Distal Unknown	29 (31.5) 63 (68.5) 0 (0.0)	6 (42.9) 7 (50.0) 1 (7.1)	0.074
Lauren subtype Intestinal Non-intestinal	63 (68.5) 29 (31.5)	14 (100.0) 0 (0.0)	0.010
Histological grade Low Grade (G1–G2) High degree (G3–G4)	51 (55.4) 41 (44.6)	13 (92.9) 1 (7.1)	0.008
Tumour invasion (T) T1–T2 T3–T4	33 (35.9) 59 (64.1)	10 (71.4) 4 (28.6)	0.018
Lymph node involvement (N) N0 N1-3	43 (46.7) 49 (53.3)	5 (35.7) 9 (64.3)	0.568
Lymphovascular invasion No Yes	42 (45.7) 50 (54.3)	6 (42.9) 8 (57.1)	0.539
Perineural invasion No Yes	46 (50.0) 46 (50.0)	6 (42.9) 8 (57.1)	0.776
Adjuvant therapy No Yes	38 (41.3) 54 (58.7)	8 (57.1) 6 (42.9)	0.141
